# LAMTOR1 regulates dendritic lysosomal positioning in hippocampal neurons through TRPML1 inhibition

**DOI:** 10.3389/fncel.2024.1495546

**Published:** 2024-11-22

**Authors:** Jiandong Sun, Weiju Lin, Xiaoning Hao, Michel Baudry, Xiaoning Bi

**Affiliations:** ^1^College of Osteopathic Medicine of the Pacific, Western University of Health Sciences, Pomona, CA, United States; ^2^College of Dental Medicine, Western University of Health Sciences, Pomona, CA, United States

**Keywords:** calcium, dynein, LAMP2, mTOR, Ragulator, lysosomal trafficking

## Abstract

Intracellular lysosomal trafficking and positioning are fundamental cellular processes critical for proper neuronal function. Among the diverse array of proteins involved in regulating lysosomal positioning, the Transient Receptor Potential Mucolipin 1 (TRPML1) and the Ragulator complex have emerged as central players. TRPML1, a lysosomal cation channel, has been implicated in lysosomal biogenesis, endosomal/lysosomal trafficking including in neuronal dendrites, and autophagy. LAMTOR1, a subunit of the Ragulator complex, also participates in the regulation of lysosomal trafficking. Here we report that LAMTOR1 regulates lysosomal positioning in dendrites of hippocampal neurons by interacting with TRPML1. LAMTOR1 knockdown (KD) increased lysosomal accumulation in proximal dendrites of cultured hippocampal neurons, an effect reversed by TRPML1 KD or inhibition. On the other hand, TRPML1 activation with ML-SA1 or prevention of TRPML1 interaction with LAMTOR1 using a TAT-decoy peptide induced dendritic lysosomal accumulation. LAMTOR1 KD-induced proximal dendritic lysosomal accumulation was blocked by the dynein inhibitor, ciliobrevin D, suggesting the involvement of a dynein-mediated transport. These results indicate that LAMTOR1-mediated inhibition of TRPML1 is critical for normal dendritic lysosomal distribution and that release of this inhibition or direct activation of TRPML1 results in abnormal dendritic lysosomal accumulation. The roles of LAMTOR1-TRPML1 interactions in lysosomal trafficking and positioning could have broad implications for understanding cognitive disorders associated with lysosomal pathology and calcium dysregulation.

## 1 Introduction

Intracellular trafficking of various cell organelles is a complex and tightly regulated process, which ensures their proper delivery and distribution within eukaryotic cells. For neurons, such trafficking is of utmost importance to maintain normal synaptic function and neuronal health. Lysosomes and late endosomes (hereafter referred to as lysosomes) have been shown to not only function as primary degradation and recycling centers but also to provide platforms for and coordinate energy sensing and regulation of various cell signaling pathways, e.g., signaling via the mechanistic target of rapamycin complex 1 (mTORC1) (Nada et al., 2009; Sancak et al., 2010; Zhang et al., 2014). They also serve as a small but important intracellular Ca^2+^ source with multiple channels/transporters regulating Ca^2+^ release and refilling (Morgan et al., 2011; Xu and Ren, 2015). Thus, lysosomal positioning within different neuronal compartments is critical for maintaining cellular homeostasis.

Lysosomal properties and functions vary based on their intracellular localization. Perinuclear lysosomes are more acidic and contain more mature hydrolases, as compared to peripheral lysosomes, and the latter may play more important roles in intercellular communications (Jia and Bonifacino, 2019; Korolchuk et al., 2011). In most cell types, lysosomes are transported along microtubules either anterogradely by coupling to plus-end directed motor proteins (kinesins), or retrogradely by coupling to minus-end directed motor proteins (the dynein-dynactin complex) (Pu et al., 2016). The intracellular transport of lysosomes is influenced by many factors, including lysosomal Ca^2+^ level (Li X. et al., 2016). It has recently been shown that synaptic activity induces lysosome recruitment into dendritic spines (Goo et al., 2017).

One of the key regulators of lysosomal positioning in dendrites is the Transient Receptor Potential Mucolipin 1 (TRPML1) protein, which is predominantly localized in late endosomes and lysosomes. Mutations in TRPML1 have been linked to lysosomal storage disorders, such as mucolipidosis type IV (Sun et al., 2000). TRPML1 mediates lysosomal Ca^2+^ release, which regulates diverse physiological functions, including lysosomal exocytosis (Medina et al., 2011; Park et al., 2016; Samie et al., 2013), membrane repair (Cheng et al., 2015), autophagy (Medina and Ballabio, 2015), nutrient sensing (Wang et al., 2015), oxidative stress sensing (Zhang et al., 2016), lysosomal motility, and lysosomal tubulation and reformation (Li X. et al., 2016). TRPML1 activity is regulated by pH (Raychowdhury et al., 2004), and its response to its endogenous agonist, PI(3,5)P2, a phosphoinositide enriched in lysosomes, differs in lysosomes localized in perinuclear vs. peripheral areas of cells (Dong et al., 2010; Zhang et al., 2012).

The lysosome-localized Ragulator complex, consisting of LAMTOR1 (p18), LAMTOR2 (p14), LAMTOR3 (MP1), LAMTOR4 (C7orf59), and LAMTOR5 (HBXIP), plays a pivotal role in recruiting and activating mTORC1 (Bar-Peled et al., 2012; Nada et al., 2009; Sancak et al., 2010). While previous research has demonstrated its interaction with the BLOC-1-related complex (BORC) in regulating lysosome positioning and axonal anterograde lysosome trafficking (Farias et al., 2017; Filipek et al., 2017; Pu et al., 2017), our group has established that it also modulates TRPML1 activation, promoting lysosomal motility in dendrites of hippocampal neurons (Sun et al., 2022). The present study investigated the effects of LAMTOR1-TRPML1 interaction on lysosomal positioning in dendrites of hippocampal neurons. We show here that LAMTOR1 regulates lysosomal trafficking and positioning by inhibiting TRPML1 activity.

## 2 Materials and methods

### 2.1 Animals

Animal experiments were conducted in accordance with the principles and procedures of the National Institutes of Health Guide for the Care and Use of Laboratory Animals. All protocols were approved by the Institutional Animal Care and Use Committee of Western University of Health Sciences. Original mice were obtained from The Jackson Laboratory, strain B6129SF2/J (Stock No:101045), and a breeding colony was established. Mice, housed in groups of two to three per cage, were maintained on a 12-h light/dark cycle with food and water *ad libitum*.

### 2.2 Hippocampal neuronal cultures

Hippocampal neurons were prepared from E18 mouse embryos as described (Sun et al., 2015). Briefly, hippocampi were dissected and digested with papain (2 mg/ml, Sigma) for 30 min at 37°C. Dissociated cells were plated onto poly-L-lysine-coated 6-well plate at a density of 6–10 × 10^4^ cells/cm^2^ or confocal dishes/coverslips in 24-well plate at a density of 6–10 × 10^3^ cells/cm^2^ in Neurobasal medium (Gibco) supplemented with 2% SM1 (STEMCELL) and 2 mM glutamine and kept at 37°C under 5% CO_2_. Half of the culture medium was replaced with fresh culture medium at DIV4 and then every 7 days.

### 2.3 Transfection and AAV infection

Cultured hippocampal neurons were transfected with Accell LAMTOR1 siRNA or Accell Non-targeting siRNA (GE Dharmacon) at DIV 4; cultured neurons were used for Western blot analysis 96 h after the transfection.

Cultured hippocampal neurons were infected with LAMTOR1 shRNA AAV, LAMTOR2 shRNA AAV, or scrambled shRNA AAV (Vector Biolabs) with GFP lentiviral vector (Santa Cruz Biotechnology) at DIV 7, and 24 h after infection, 2/3 medium was replaced with fresh medium. For LAMTOR1 rescue experiments, LAMTOR1 shRNA AAV-infected neurons were infected with RNAi-resistant LAMTOR1 AAV (VectorBuilder) at DIV14, and neurons were analyzed at DIV21. For TRPML1 KD experiment, neurons were co-transfected with Accell TRPML1 siRNA or control siRNA (GE Dharmacon) at DIV 17, and neurons were analyzed at DIV21.

### 2.4 Antibodies, chemicals, and DNA constructs

Antibodies, chemicals, and plasmids used in this study are listed in Table 1. The following peptides were synthesized by ABI Scientific: TAT (YGRKKRRQRRR), and TAT-2031-m (mouse, YGRKKRRQRRRKLLLDPSSTPTK).

**TABLE 1:** Antibodies, chemicals, and plasmids used in this study.

Reagent or resource	Source	Identifier
**Antibodies**		
Rabbit monoclonal anti-LAMTOR1	Cell Signaling Technology	Cat#8975
Rabbit polyclonal anti-LAMTOR1	Sigma-Aldrich	Cat#HPA002997
Rabbit monoclonal anti-LAMTOR2	Cell Signaling Technology	Cat#8145
Rat monoclonal anti-LAMP2	Abcam	Cat#ab13524
Goat polyclonal anti-Cathepsin B	R&D Systems	Cat#AF965
Rabbit polyclonal anti-TRPML1	Alomone	Cat#ACC-081
Rat monoclonal anti-Rab11	Abcam	Cat#ab95375
Rabbit polyclonal anti-GFP	Abcam	Cat#ab290
Chicken polyclonal anti-GFP	Thermo Fisher Scientific	Cat#A10262
Mouse monoclonal anti-GAPDH (clone 6C5)	EMD Millipore	Cat#MAB374
Mouse monoclonal anti-β-actin (clone AC-15)	Sigma-Aldrich	Cat#A5441
Goat anti-rabbit IgG IRDye^®^ 680RD	LI-COR Biosciences	Cat#926-68071
Goat anti-mouse IgG IRDye^®^ 800CW	LI-COR Biosciences	Cat#926-32210
Goat anti-rat IgG IRDye^®^ 680RD	LI-COR Biosciences	Cat#926-68076
Donkey anti-mouse IgG AlexaFluor 594	Invitrogen	Cat#A-21203
Goat anti-rabbit IgG AlexaFluor 488	Invitrogen	Cat#A-11008
Goat anti-rabbit IgG AlexaFluor 594	Invitrogen	Cat#A-11037
Donkey anti-rat IgG AlexaFluor 594	Invitrogen	Cat#A-21209
Goat anti-chicken IgY AlexaFluor 488	Invitrogen	Cat#A-11039
Goat anti-rabbit IgG AlexaFluor 633	Invitrogen	Cat#A-21070
Donkey anti-goat IgG AlexaFluor Plus 594	Invitrogen	Cat#A-32758
**Bacterial and virus strains**		
GFP Control Lentiviral Particles	Santa Cruz Biotechnology	Cat#sc-108084
Scrambled AAV9 shRNA Control Virus	Vector Biolabs	Cat#7045
LAMTOR1 AAV9 shRNA Virus	Vector Biolabs	Custom
LAMTOR2 AAV9 shRNA Virus	Vector Biolabs	Cat#shAAV-270773
Scrambled AAV9 shRNA Control Virus	VectorBuilder	Custom
LAMTOR1 AAV9 shRNA Virus	VectorBuilder	Custom
RNAi-resistant LAMTOR1 AAV9 Virus	VectorBuilder	Custom
**Chemicals, peptides, and recombinant proteins**		
ML-SA1	Tocris	Cat#4746
ML-SI1	Alfa Aesar	Cat#J67425
ML-SI1	Sigma	Cat#G1421
Ciliobrevin D	EMD Millipore	Cat#250401
Torin 1	Tocris	Cat#4247
TAT	ABI Scientific	Custom
TAT-2031-mouse	ABI Scientific	Custom
Lipofectamine 2000	Invitrogen	Cat#11668019
LysoTracker Red DND-99	Invitrogen	Cat#L7528
**Oligonucleotides**		
SMARTpool Accell Mouse LAMTOR1 siRNA	Dharmacon	Cat#E-048359-01
SMARTpool Accell Mouse TRPML1 siRNA	Dharmacon	Cat#E-044469-00
Accell Non-targeting Control Pool	Dharmacon	Cat#D-001910-10

Further information and requests for resources and reagents should be directed to and will be fulfilled by Corresponding author, Xiaoning Bi (xbi@westernu.edu).

### 2.5 Live cell imaging

LysoTracker Red DND-99 (100 nM) was dissolved in culture medium and loaded into cells for 1 h before imaging. Live-cell imaging was performed in complete medium using a Zeiss LSM880 AiryScan confocal microscope equipped with a Plan-Apochromat 63 × /1.4 oil immersion objective, and an environmental chamber set at 37°C and 5% CO_2_. Images were acquired over a period of 1 min at 1-s intervals and processed by using software ZEN (Zeiss) including brightness adjustment, conversion of images to movies, and kymograph generation.

### 2.6 Western blot analysis

Neurons were lysed with CHAPS lysis buffer (Tris-HCl 25 mM pH 7.4, NaCl 150 mM, 1 mM EDTA, 0.5% CHAPS, 5% glycerol and a protease inhibitor cocktail). Protein concentrations were determined with a BCA protein assay kit (Pierce). Western blots were performed according to published protocols (Sun et al., 2015). Briefly, samples were separated by SDS-PAGE and transferred onto a PVDF membrane (Millipore). After blocking with 3% BSA for 1 h, membranes were incubated with specific antibodies overnight at 4°C followed by incubation with IRDye secondary antibodies for 2 h at room temperature. Antibody binding was detected with the Odyssey^®^ family of imaging systems.

### 2.7 Immunofluorescence

Cultured hippocampal neurons were fixed in 2% paraformaldehyde (PFA)/10% sucrose for 15 min at 37°C, transferred to 0.05% Triton X-100/PBS for 5 min at 4°C, and then 0.02% Tween-20/PBS for 2 min at 4°C. Coverslips were washed twice with ice cold PBS and incubated 1 h in 3% BSA/PBS at room temperature. Cells were incubated with anti-LAMTOR1 (1:200, Sigma) and anti-LAMP2 (1:200 respectively in 3% BSA/PBS overnight at 4°C). For staining of cathepsin B, EEA1, and Rab11, neurons were fixed in 2% paraformaldehyde (PFA)/10% sucrose for 15 min at 37°C, incubated with a blocking buffer (0.4% saponin, 1% BSA, and 5% goat or donkey serum in PBS) for 1 h and then incubated with anti-cathepsin B (1:20), anti-EEA1 (1:500), and anti-Rab11 (1:100) respectively diluted in the incubation buffer (0.1% saponin, 1% BSA, and 5% goat or donkey serum in PBS) overnight at 4°C. After three washes in PBS, the cells were incubated with an appropriate Alexa Fluor–conjugated secondary antibody for 2 h at room temperature. Coverslips were then washed four times with PBS and mounted on glass slides using VECTASHIELD mounting medium with DAPI (Vector Laboratories). Images were acquired using a Zeiss LSM 880 confocal laser-scanning microscope in Airyscan mode. The staining was visualized in GFP-expressed neurons.

### 2.8 Image analysis and quantification

Images for all groups in a particular experiment were obtained using identical acquisition parameters and analyzed using Zen (Zeiss) or ImageJ (NIH) software. In all cases the experimenter was blind regarding the identity of the samples during acquisition and analysis. For quantification of LAMP2 or cathepsin B distribution along the proximal dendrites (60 μm from the origin of the dendrites), images were analyzed using Fiji. Dendrite segments were selected by segmented line tool with the line width set at 50–75, and then analyzed by Plot Profile. The mean fluorescence intensity of LAMP2 or cathepsin B staining was averaged every 5 μm.

### 2.9 Statistical analysis

All data are expressed as means ± SEM. To compute *p*-values, unpaired Student’s *t*-test, and two-way ANOVA with Tukey’s or Sidak’s post-test were used (GraphPad Prism 6), as indicated in figure legends. The level of statistical significance was set at *P* < 0.05.

## 3 Results

### 3.1 LAMTOR1 regulates lysosomal positioning in dendrites of cultured hippocampal neurons

**FIGURE 1:**
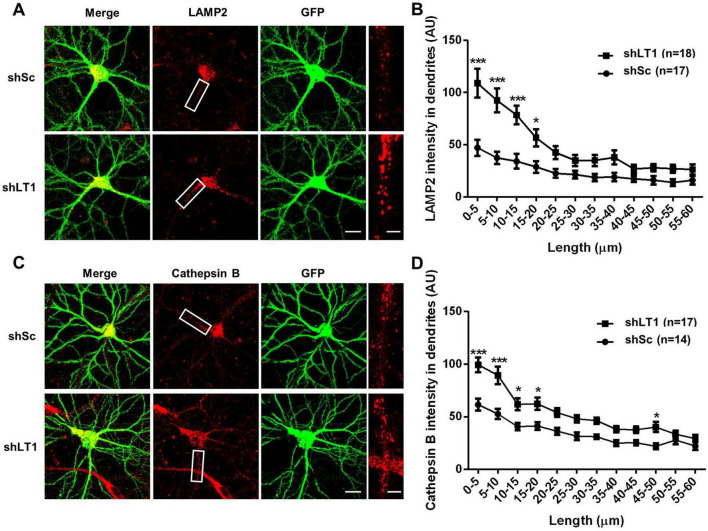
LAMTOR1 regulates lysosomal positioning in dendrites of cultured hippocampal neurons. See also Supplementary Figures 1–3. **(A)** Images of cultured hippocampal neurons stained for LAMP2 (red). Neurons were infected with an shRNA AAV directed against LAMTOR1 (shLT1) or a scrambled shRNA control (shSc) with GFP co-expression before being processed for immunofluorescence assay and imaging. Insets: enlarged dendrites. Scale bar: 20 μm, and 5 μm in insets. **(B)** Quantification of lysosome distribution along the dendrites, as shown in **(A)**. Results are Means ± SEM of 17–18 neurons from 6 independent experiments, *p < 0.05, ***p < 0.001, as compared with shSc, two-way ANOVA with Sidak’s post-test. **(C)** Images of cultured hippocampal neurons stained for cathepsin B (red). Neurons were infected as described in **(A)** before being processed for immunofluorescence assay and imaging. Insets: enlarged dendrites. Scale bar: 20 μm, and 5 μm in insets. **(D)** Quantification of lysosome distribution along the dendrites, as shown in **(C)**. Results are Means ± SEM of 14–17 neurons from 3 independent experiments, *p < 0.05, ***p < 0.001, as compared with shSc, two-way ANOVA with Sidak’s post-test.

We previously reported that LAMTOR1 restricts dendritic lysosomal trafficking through inhibition of TRPML1, since LAMTOR1 knockdown (KD) resulted in enhanced lysosomal motility and trafficking in a TRPML1-dependent manner (Sun et al., 2022). In the current study, we tested whether LAMTOR1 KD could affect lysosomal positioning in dendrites of cultured hippocampal neurons. Cultured hippocampal neurons were infected on day 7 *in vitro* (DIV7) with a LAMTOR1 shRNA or a scrambled shRNA and were tested 14 days later. In scrambled shRNA-infected neurons, lysosomes were found mostly in cell bodies, often clustered around nuclei, with a few distributed in dendrites, which is consistent with the literature (Schwenk et al., 2014; Yap et al., 2018). In contrast, increased numbers of lysosomes accumulated in proximal dendrites following LAMTOR1 KD (Figure 1A). Quantitative analysis revealed a significant increase in the density of LAMP2-positive lysosomes in proximal dendrites, up to 20 μm from the origin of the dendrites (Figure 1B). Immunofluorescence staining with cathepsin B, a lysosomal protease, showed that the density of cathepsin B-positive lysosomes similarly increased in proximal dendrites (Figures 1C, D). We confirmed LAMTOR1 KD efficiency by immunofluorescence. As previously reported (Sun et al., 2022), the LAMTOR1 shRNA significantly reduced LAMTOR1 expression, and this effect was reversed by the expression of a shRNA-resistant LAMTOR1 construct (Supplementary Figure 1A). We also confirmed that LAMTOR1 KD markedly increased the motility of LysoTracker-labeled lysosomes (Supplementary Figure 1B), a result consistent with our previously published data (Sun et al., 2022). In contrast to lysosomal positioning, LAMTOR1 KD had no effects on the distribution of early endosomes and recycling endosomes labeled with EEA1 and Rab11, respectively (Supplementary Figures 2A, B). These results are consistent with our previous finding that LAMTOR1 KD did not affect the trafficking of transferrin-labeled endosomes.

**FIGURE 2:**
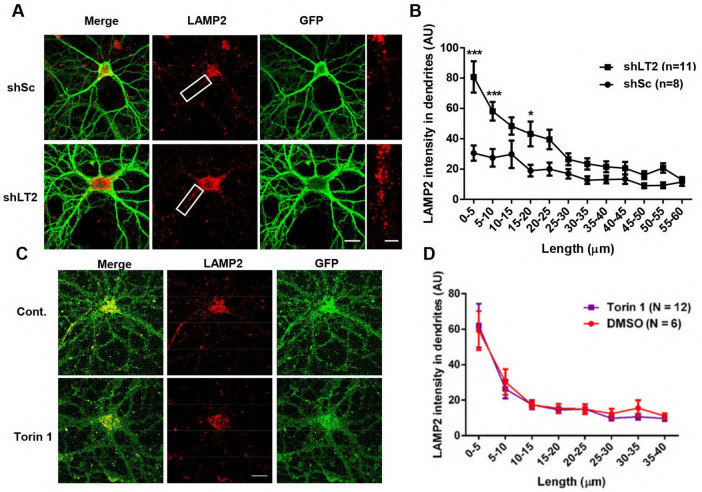
LAMTOR2 regulates lysosomal positioning in dendrites of cultured hippocampal neurons, while mTORC1 inhibition has no effect. See also Supplementary Figure 2. **(A,B)** Effects of LAMTOR2 KD on dendritic lysosomal positioning. **(A)** Images of cultured hippocampal neurons stained for LAMP2 (red). Neurons were infected with an shRNA AAV directed against LAMTOR2 (shLT2) or a scrambled shRNA control (shSc) with GFP co-expression before being processed for immunofluorescence assay and imaging. Insets: enlarged dendrites. Scale bar: 20 μm, and 5 μm in insets. **(B)** Quantification of lysosome distribution along the dendrites, as shown in **(A)**. Results are Means ± SEM of 8–11 neurons from 3 independent experiments, *p < 0.05, ***p < 0.001, as compared with shSc, two-way ANOVA with Sidak’s post-test. **(C,D)** Effects of mTORC1 inhibition on dendritic lysosomal positioning. **(C)** Images of cultured hippocampal neurons stained for LAMP2 (Red) and GFP. Neurons were treated with vehicle control or with Torin 1 (250 nM) for 4 h before being processed for immunofluorescence assay and imaging. Scale bar: 20 μm. **(D)** Quantification of lysosome distribution along the dendrites, as shown in **(C)**. Data are represented as Means ± SEM. *N* = 6–12 neurons from 3 independent experiments, two-way ANOVA with Sidak’s post-test.

We next knocked down LAMTOR2, another member of the Ragulator complex, using a LAMTOR2 shRNA AAV in cultured hippocampal neurons. LAMP2-positive lysosomes in LAMTOR2 KD neurons showed a distribution similar to that found in LAMTOR1 KD neurons (Figures 2A, B). Since the Ragulator complex plays crucial roles in mTORC1 activation and mTORC1 has been shown to regulate not only lysosomal biogenesis but also TRPML1 activity, we tested the effect of Torin 1 (250 nM for 4 h), an mTOR inhibitor, on the distribution of LAMP2-positive particles. Image analysis showed that there was no significant difference in the distribution of lysosomes in dendrites of Torin 1-treated neurons, as compared to vehicle-treated neurons (Figures 2C, D). These results indicate that lysosome positioning in dendrites of hippocampal neurons depends on the Ragulator rather than on mTORC1 activity.

LAMTOR1 KD efficiency and the effects of LAMTOR1 down-regulation on some proteins associated with other organelles were also determined by Western blot analysis of whole homogenate and lysosomal fraction of cultured neurons (Supplementary Figure 3). Lysosomal fractionation showed that, while there was a clear enrichment of LAMTOR1 in the lysosomal fraction, the levels of COXIV (a mitochondrial marker) and EEA1 (an early endosomal marker) were dramatically reduced in this fraction, as compared to the amount in whole homogenates. LAMTOR1 shRNA-mediated KD markedly reduced its levels in both homogenate and lysosomal fractions. LAMP2 was present in whole homogenate and lysosomal fractions, and LAMTOR1 KD slightly increased its amount in the lysosomal fraction; the borderline increase in LAMP2 levels is consistent with what we previously published (Sun et al., 2022). Noticeably, significant amounts of the motor proteins, kinesin and dynein, the synaptic marker, synaptophysin, the tyrosinated and detyrosinated forms of tubulin (microtubule proteins), and the recycling endosome marker Rab11, were also found in the lysosomal fraction, although their levels were not significantly affected by LAMTOR1 KD. These results indicate that LAMTOR1 KD-induced lysosomal accumulation in proximal dendrites of hippocampal neurons is unlikely due to changes in the amount of either motor proteins or tyrosinated/detyrosinated tubulin, or to a marked increase in lysosomal biogenesis.

### 3.2 The effects of LAMTOR1 KD on lysosomal trafficking and positioning depend on TRMPL1 activation

**FIGURE 3:**
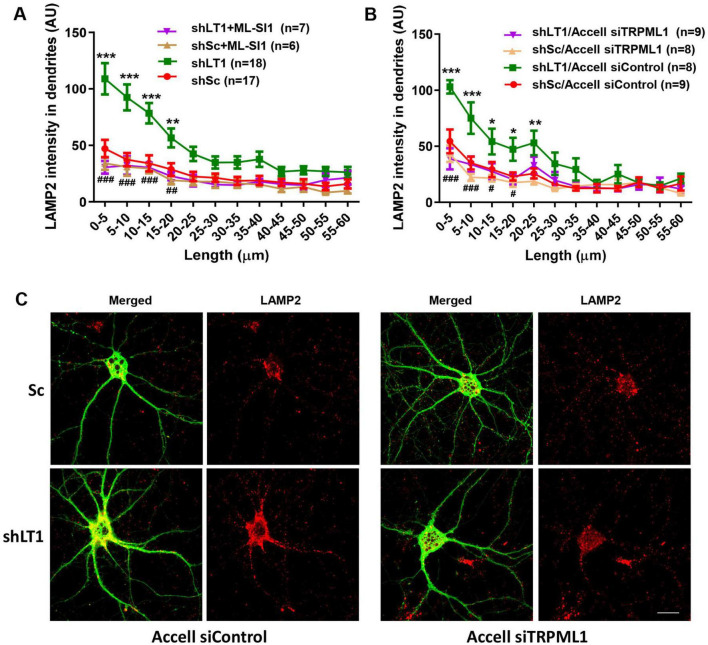
The effects of LAMTOR1 KD on lysosomal positioning depend on TRMPL1-mediated Ca^2+^ release. **(A)** The TRPML1 inhibitor, ML-SI1, blocked the effects of LAMTOR1 KD on dendritic lysosomal positioning. Cultured hippocampal neurons were infected with AAV expressing either a LAMTOR1 shRNA (shLT1) or a scrambled shRNA (shSc); they were then treated with vehicle control or ML-SI1 before being processed for LAMP2 staining. Results are Means ± SEM of 6–18 neurons from 3 to 6 independent experiments, **p < 0.01, ***p < 0.001, as compared with shSc (green line compared to red line), ##*p* < 0.01, ###*p* < 0.001, as compared with shLAMTOR1 (purple line compared to green line), two-way ANOVA with Tukey’s post-test. **(B)** TRPML1 KD blocked the effects of LAMTOR1 KD on dendritic lysosomal positioning. Cultured hippocampal neurons were infected with shLAMTOR1 (shLT1) or shSc AAV and Accell TRPML1 siRNA or control siRNA; they were then being processed for LAMP2 staining. Results are Means ± SEM of 8–9 neurons from 3 independent experiments, *p < 0.05, **p < 0.01, ***p < 0.001, as compared with shSc/Accell siControl (green line compared to red line), #*p* < 0.05, ###*p* < 0.001, as compared with shLAMTOR1/Accell siControl (purple line compared to green line), two-way ANOVA with Tukey’s post-test. Note that the data for shSc and shLT1 in **(A)** are the same as those shown in Figure 1B. **(C)** Representative images of with shLT1 or shSc AAV and Accell TRPML1 siRNA or control siRNA and stained with LAMP2 and GFP. Scale bar: 20 μm.

To determine whether TRPML1 activation could contribute to LAMTOR1 KD-induced changes in lysosomal positioning, we first used a TRPML1 inhibitor, ML-SI1. Treatment of cultured hippocampal neurons with 20 μM ML-SI1 for 2 h blocked LAMTOR1 KD-induced changes in lysosomal positioning (Figure 3A). Similar results were obtained with Accell siRNA-mediated TRPML1 KD (Figures 3B, C), which confirmed that LAMTOR1 KD induced lysosomal accumulation in proximal dendrites in a TRPML1-dependent manner. Neither TRPML1 inhibitor nor Accell siRNA-mediated TRPML1 KD affected lysosomal dendritic distribution in control neurons.

**FIGURE 4:**
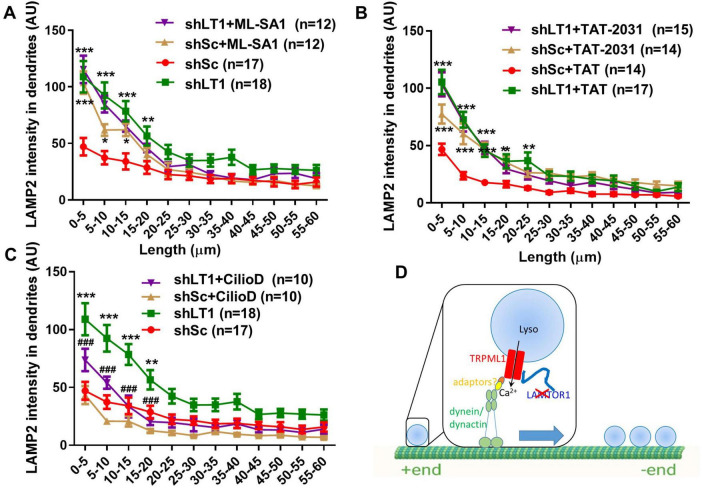
TRPML1 activation by ML-SA1 or blockade of LAMTOR1/TRPML1 inhibitory interaction reproduced the effects of LAMTOR1 KD on lysosome positioning in a dynein-dependent manner. **(A)** The TRPML1 activator ML-SA1 induced lysosomal accumulation in proximal dendrites. Neurons were infected with shLAMTOR1 (shLT1) or shSc AAV and treated with either a vehicle control or ML-SA1 before being processed for LAMP2 staining. Results are Means ± SEM of 12–18 neurons from 3 to 6 independent experiments, *p < 0.05, **p < 0.01, ***p < 0.001, as compared with shSc. **(B)** Disruption of LAMTOR1-TRPML1 interaction induced lysosomal accumulation in proximal dendrites. Neurons were infected with shLAMTOR1 (shLT1) or shSc AAV, treated with TAT or TAT-2031 (10 μM) for 3 h, before being processed for LAMP2 staining. Results are Means ± SEM of 14–17 neurons from 3 independent experiments, *p < 0.05, **p < 0.01, ***p < 0.001, as compared with shSc+TAT. **(C)** Inhibition of dynein blocked LAMTOR KD induced lysosomal accumulation. Neurons were infected with shLAMTOR1 (shLT1) or shSc AAV, treated with vehicle control or Ciliobrevin D (CilioD), before being processed for LAMP2 staining. Results are Means ± SEM of 10–18 neurons from 3 independent experiments, **p < 0.01, ***p < 0.001, as compared with shSc, ###*p* < 0.001 compared with shLAMTOR1. *P*-values were derived by two-way ANOVA with Tukey’s post-test. Note that the data for shSc and shLAMTOR1 in **(A,C)** are the same as those shown in Figure 1B. **(D)** Diagram showing that deletion of LAMTOR1 or disruption of LAMTOR1/TRPML1 interaction (red X) enhances dynein-mediated retrograde lysosomal trafficking and proximal dendrite positioning.

Next, we analyzed whether TRPML1 activation was sufficient to alter lysosomal positioning. When scrambled shRNA-treated neurons were treated with the TRPML1 activator, ML-SA1 (10 μM), for 42 h, lysosomal accumulation in proximal dendrites was also observed (Figure 4A). ML-SA1 treatment had no effect on lysosomal positioning in LAMTOR1 shRNA-transfected neurons (Figure 4A), suggesting an occlusion effect. These experiments indicate that LAMTOR1 regulates lysosome motility and distribution by inhibiting TRPML1 channel function.

We previously showed that LAMTOR1 inhibits TRPML1 through its N-terminal domain interaction with TRPML1, and we designed a decoy peptide (TAT-2031) to block this interaction (Sun et al., 2022). We analyzed the effects of treating cultured hippocampal neurons with TAT-2031 (10 μM) or its control TAT peptide (10 μM) for 3 h on dendritic lysosomal positioning. Treatment with TAT-2031, but not the control peptide, increased lysosomal density in proximal dendrites of scrambled shRNA-infected neurons, while it had no effect in LAMTOR1 KD neurons (Figure 4B), which also suggests an occlusion effect.

TRPML1 activation promotes retrograde transport of lysosomes through the activation of cytoplasmic dynein motors (Li X. et al., 2016). We therefore treated cultured hippocampal neurons with the dynein inhibitor ciliobrevin D (20 μM) for 1.5 h, as previously described (Ayloo et al., 2017). Ciliobrevin D treatment significantly decreased lysosomal density in proximal dendrites of LAMTOR1 KD neurons, while it had no significant effect in scrambled shRNA-infected neurons, although it slightly reduced the values in the 5 to 20 μm range from the origin of the dendrites (Figure 4C).

All together, these results indicate that LAMTOR1 regulates lysosomal positioning by inhibiting TRPML1. When this inhibition is relieved, by either LAMTOR KD or treatment with the TAT-2031 peptide, or when TRPML1 is activated by ML-SA1, lysosomal Ca^2+^ release through TRPML1 is increased (as we previously demonstrated (Sun et al., 2022)), which results in the activation of the retrograde motor dynein, facilitation of lysosomal transport, and lysosomal accumulation in proximal dendrites and eventually in cell bodies as well (Figure 4D).

## 4 Discussion

We previously showed that LAMTOR1 KD enhanced dendritic lysosomal motility, which was dependent on TRPML1-mediated lysosomal Ca^2+^ release. We demonstrated that LAMTOR1 inhibited TRPML1 Ca^2+^ release through a direct interaction between its N-terminal domain and TRPML1. We further showed that LAMTOR1 KD impaired LTP induction and enhanced LTD expression, and both effects were TRPML1-dependent. Furthermore, LAMTOR1 KD resulted in fear conditioning impairment, which was reversed by treatment with the TRPML1 inhibitor, ML-SI1 (Sun et al., 2022). Results from the current study expand these findings and indicate that LAMTOR1 KD or preventing its interaction with TRPML1 results in lysosomal accumulation in proximal dendrites of hippocampal pyramidal neurons. These effects are also dependent on TRPML1 activation and involve the retrograde motor protein dynein.

TRPML1 plays a critical role in membrane trafficking, lysosomal exocytosis, and lysosomal biogenesis (Li X. et al., 2016; Medina et al., 2011; Medina et al., 2015). The robust effects of LAMTOR1 KD on lysosomal positioning are consistent with our published data showing that LAMTOR1 inhibits TRPML1-mediated Ca^2+^ release. In support of this mechanism, we showed that genetic or pharmacological inhibition of TRPML1 fully restored the altered dendritic lysosomal positioning induced by LAMTOR1 KD, while TRPML1 activation by ML-SA1 reproduced the effects of LAMTOR1 KD, indicating that these lysosomal changes are due to the relief of the inhibitory effect of LAMTOR1 on TRPML1. We also confirmed the roles of the interaction between LAMTOR1 and TRPML1 by using a peptide specifically targeting the TRPML1 binding domain in LAMTOR1. The effects of LAMTOR2 KD on lysosomal positioning are most likely due to its impact on Ragulator stability, thereby affecting LAMTOR1-mediated inhibition of TRPLM1, as previously reported (de Araujo et al., 2013).

In addition to direct interactions between LAMTOR1 and TRPML1, LAMTOR1 could affect lysosomes through the activation of mTORC1. TRPML1 channel is inhibited by mTOR-mediated phosphorylation (Onyenwoke et al., 2015), although this is still debated (Li R. J. et al., 2016; Yang et al., 2019). However, inhibition of mTORC1 by Torin 1 had no effect on lysosome positioning, in agreement with previous observations (Pu et al., 2017). Along this line, endosome/lysosome recording has shown that Torin 1 did not activate *I*_TRPML1_ (Zhang et al., 2019). Therefore, the regulation of TRPML1 by LAMTOR1 is independent of mTORC1 activity.

Compared to lysosomal trafficking in axons, relatively little is known regarding lysosomal trafficking in dendrites (Farias et al., 2017; Goo et al., 2017; Gowrishankar et al., 2017; Yap et al., 2018). We previously showed that TRPML1-mediated Ca^2+^ release facilitates both centripetal and centrifugal lysosomal trafficking in a dynein-dependent manner, which is most likely due to the complex microtubule arrangement in dendrites of mature neurons (Baas et al., 1988; Tas et al., 2017). Nevertheless, we showed here that LAMTOR1 KD resulted in lysosomal accumulation in proximal dendrites, which would be consistent with increased retrograde lysosomal transport. These results agree with previous reports investigating dendritic endosomal/lysosomal trafficking, which showed that dynein favors retrograde trafficking, while kinesin facilitates anterograde trafficking even in dendrites (Ayloo et al., 2017; Lipka et al., 2016). One possible explanation for missing this effect in our previous live lysosomal recording could be that the time frame we used was not long enough to reveal this retrograde bias.

Surprisingly, treatment with the dynein inhibitor, ciliobrevin D, did not alter lysosomal trafficking in control/scrambled shRNA-treated neurons, although there was a slight but not significant decrease in lysosomal density in dendritic fragments from 5 to 20 μm from the origin of the dendrites. It is possible that at the incubation time and concentration of ciliobrevin D we used only significantly affected dynein-mediated retrograde lysosomal trafficking initiated by TRPML1 activation. It is also possible that other motor proteins are involved in dendritic lysosomal trafficking under basal conditions. The fact that ciliobrevin D treatment only partially blocked the effects of LAMTOR1 KD-induced lysosomal accumulation also supports the notion that other motor proteins are likely involved in dendritic lysosomal trafficking.

TRPML1-mediated lysosomal Ca^2+^ release can promote TFEB nuclear translocation leading to lysosomal biogenesis and autophagy regulation (Medina et al., 2015). It is thus conceivable that in LAMTOR1 KD neurons, TFEB is translocated to the nucleus, presumably due to mTORC1 inhibition (Martina et al., 2012; Napolitano et al., 2018) or calcineurin activation, which in turn would increase the expression of TRPML1, since TRPML1 is a target gene of TFEB (Sardiello et al., 2009). However, we previously showed that TRPML1 levels were similar in control and LAMTOR1 KD tissues (Sun et al., 2022), suggesting that lysosome biogenesis was not a dominant effect. Nevertheless, we cannot completely rule out the possibility that activation of TFEB and lysosomal biogenesis, as indicated by borderline increase in LAMP2 levels, could be facilitated at a later time point, potentially contributing to lysosomal accumulation in proximal dendrites and cell bodies in neurons.

Lysosome accumulation in primary dendrites and cell bodies is a major feature of aged neurons (Bi et al., 2003), and lysosomal dysfunction has been implicated in neurodegenerative diseases, e.g., Alzheimer’s and Parkinson’s diseases (Gowrishankar et al., 2015; Gowrishankar et al., 2017; Lie and Nixon, 2018; Moors et al., 2016). Alterations in lysosomal trafficking and positioning could have significant implications for neuronal functions since lysosomal properties, including pH, hydrolase maturation, Ca^2+^ channel activity, and nutrient sensing and mTORC1 signaling etc., are all affected by lysosomal localization along dendrites or axons. In fact, mutations in genes involved in lysosomal functions, including trafficking, have been shown to cause neurodegenerative diseases across all age groups (Lie and Nixon, 2018). TRPML1 interacts with apoptosis-linked gene 2 (ALG2) and JNK-interacting protein 4 (JIP4), thereby promoting lysosomal clustering around the microtubule organizing center in cell models of Parkinson’s disease (Date et al., 2024; Sasazawa et al., 2022; Sasazawa et al., 2024). The beneficial effects of TRPML1 have also been proposed for Alzheimer’s disease (Cen et al., 2024; Somogyi et al., 2023), seizure (Peng et al., 2024), and juvenile neuronal ceroid lipofuscinosis (Wunkhaus et al., 2024).

We recently reported that LAMTOR1 is a target of the UBE3A ubiquitin ligase, which is downregulated in Angelman syndrome (AS), thereby implicating changes in lysosomal distribution, function and mTOR signaling in the etiology of AS (Sun et al., 2018). Likewise, altered lysosomal Ca^2+^ release, including via TRPML1 channels, has also been implicated in various diseases (Sun et al., 2022). Loss of function mutations in TRPML1 result in ML-IV, an autosomal recessive lysosomal storage disease with severe neurodegeneration (Berman et al., 1974; Slaugenhaupt, 2002). Along this line, TRPML1 agonists are being developed for the treatment of neurodegenerative diseases (Genetic Engineering Biotechnology News Merck). In contrast, knockout of presenilin (PS1) in mice induces abnormal TRPML1-mediated Ca^2+^ efflux from lysosomes, thereby contributing to the pathogenesis in Familial Alzheimer’s disease (Coen et al., 2012; Lee et al., 2015). Notwithstanding the reasons for these paradoxical findings, they nevertheless underline our lack of a complete understanding of the precise functions and regulation of neuronal TRPML1. These findings also emphasize that the tight control of TRPML1-mediated Ca^2+^ release, as of other Ca^2+^ channels, is absolutely critical for normal neuronal function.

## 5 Conclusion and limitations

Our results indicate that LAMTOR1 is an endogenous negative regulator of TRPML1 channels and that LAMTOR1-mediated inhibition of lysosomal TRPML1 Ca^2+^ release is critically involved in lysosome positioning and trafficking in dendrites. These properties make the LAMTOR1/TRPML1 interaction critical for synaptic plasticity and learning and memory, and suggest that alterations in this interaction are likely involved in neurological diseases. One future direction would be to directly test the roles of LAMTOR1-mediated regulation of TRPML1 in models of neurological disorders *in vivo* and determine whether this regulation is specific to certain types of neurons and makes them vulnerable to disease-specific degeneration.

## Data Availability

The original contributions presented in this study are included in this article/Supplementary material, further inquiries can be directed to the corresponding author.
