# Supplementary material for: LAMTOR1 regulates dendritic lysosomal positioning in hippocampal neurons through TRPML1 inhibition
Source: Front Cell Neurosci. 2024 Nov 22;18:1495546. doi: 10.3389/fncel.2024.1495546 (PMC11621854; doi:10.3389/fncel.2024.1495546)
Supplement: Supplementary file 1 [file Table_1.DOCX]

**SUPPLEMENTARY INFORMATION**

**LAMTOR1 regulates dendritic lysosomal positioning in hippocampal neurons through TRPML1 inhibition**

Journal: Cellular and Molecular Life Sciences

Jiandong Sun^1^, Weiju Lin^1^, Xiaoning Hao^1^, Michel Baudry^2^, and Xiaoning Bi^1,*^

^1^College of Osteopathic Medicine of the Pacific and ^2^College of Dental Medicine, Western University of Health Sciences, Pomona, CA 91766

*Corresponding author: Xiaoning Bi, xbi@westernu.edu

**Fig. S1**


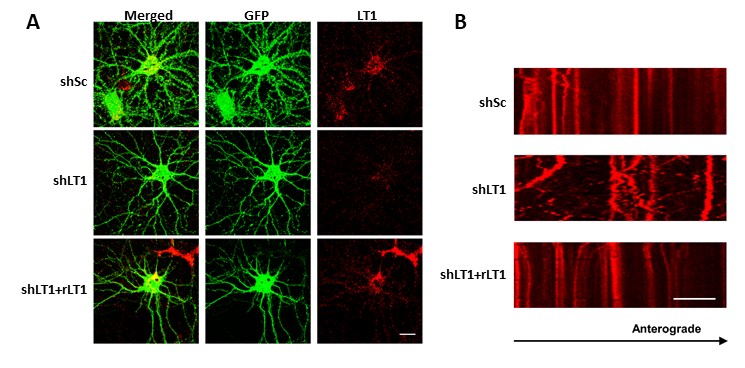


**Fig. S1: Effects of shLAMTOR1 KD on lysosome trafficking and its reversal by a shRNA-resistant LAMTOR1 in hippocampal neurons.** Related to Figure 1.

(**A**) Images of cultured hippocampal neurons immunostained for LAMTOR1 (LT1, red). Neurons were infected with an shRNA AAV directed against LAMTOR1 (shLT1) or scrambled shRNA control (shSc) with GFP co-expression with or without shRNA-resistant LAMTOR1 (rLT1) before being processed for immunofluorescence assay and imaging. Scale bar, 20 µm. (**B**) Cultured hippocampal neurons were infected as described in A; they were stained with LysoTracker (red) and imaged every second for 1 min to visualize lysosomal trafficking in dendrites. Shown are kymographs of dendritic movement of LysoTracker-labeled vesicles. Scale bar represents 5 µm.

**Fig. S2.**

**
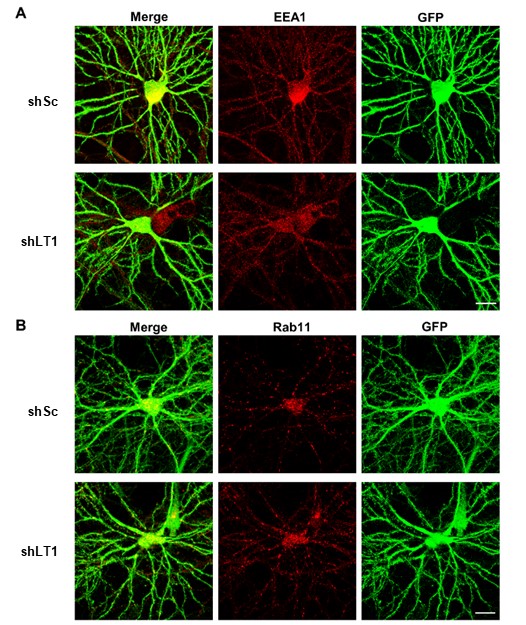
**

**Fig. S2: LAMTOR1 KD has no effects on dendritic positioning of early endosomes and recycling endosomes in cultured hippocampal neurons.** Related to Figure 1.

(**A**) Images of cultured hippocampal neurons stained for EEA1 (red). Neurons were infected with an shRNA AAV directed against LAMTOR1 (shLT1) or a scrambled shRNA control (shSc) with GFP co-expression before being processed for immunofluorescence assay and imaging. Scale bar: 20 µm. (**B**) Images of cultured hippocampal neurons stained for Rab11 (red). Neurons were infected as described in **A** before being processed for immunofluorescence assay and imaging. Scale bar: 20 µm.

**Fig. S3**


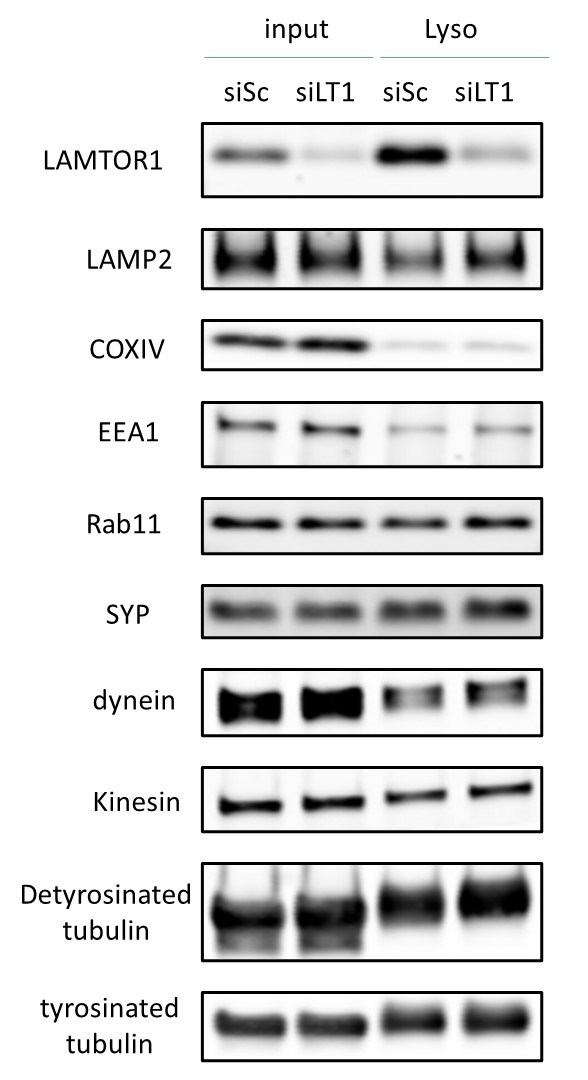


**Fig. S3: Effects of LAMTOR1 KD on various proteins.** Related to Figure 1.

Western blot analysis of LAMTOR1, LAMP2, COXIV, EEA1, Rab11, synaptophysin (SYP), dynein, kinesin, and tyrosinated/detyrosinated tubulin in whole homogenates (input) and lysosome-enriched fractions (Lyso) prepared from cultured hippocampal neurons transfected with Accell control (siSc) or LAMTOR1 siRNA (siLT1).

**
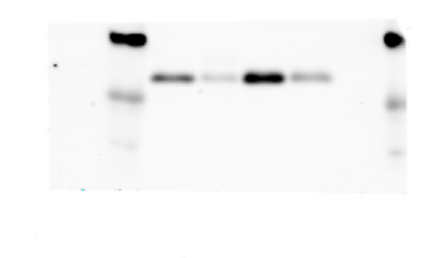

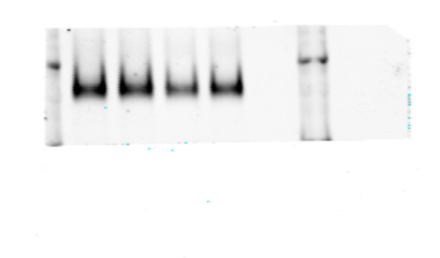

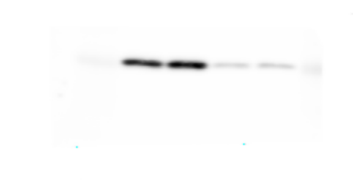
**

LAMTOR1

LAMP2

COXIV

**
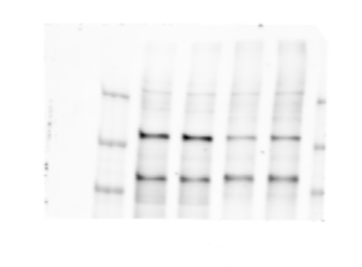
**

EEA1

**
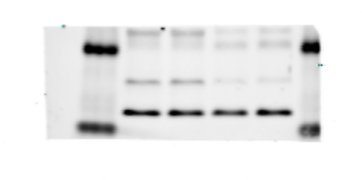

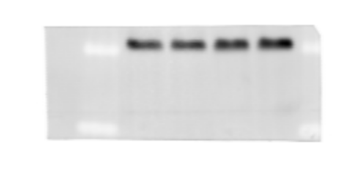
**

Rab11

SYP

**
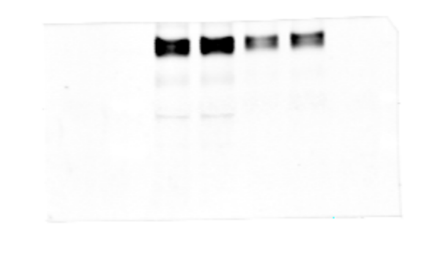
**

dynein

**
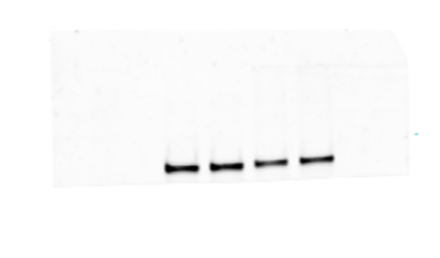

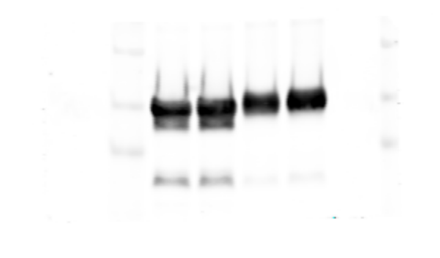
**

tyrosinated tubulin

Kinesin

Detyrosinated tubulin

**
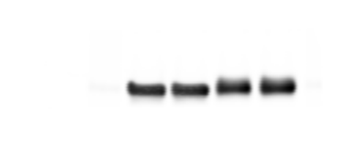
**

tyrosinated tubulin
